# Supplementary material for: Sphingomyelin Depletion from Plasma Membranes of Human Airway Epithelial Cells Completely Abrogates the Deleterious Actions of S. aureus Alpha-Toxin
Source: Toxins (Basel). 2019 Feb 20;11(2):126. doi: 10.3390/toxins11020126 (PMC6409578; doi:10.3390/toxins11020126)
Supplement: Supplementary file 1 [file toxins-11-00126-s001.pdf]

Supplementary Materials: Sphingomyelin Depletion from Plasma Membranes of Human Airway Epithelial Cells Completely Abrogates the Deleterious Actions of *S. aureus* Alpha-Toxin

Sabine Ziesemer, Nils Möller, Andreas Nitsch, Christian Müller, Achim G. Beule and Jan-Peter Hildebrandt

S1: The recombinant form of *S. aureus* Hlb has sphingomyelinase activity. To test whether recombinant hemolysin B (rHlb) had enzymatic activity as a sphingomyelinase, we used HPTLC analyses on lipid extracts from human airway epithelial cells (16HBE14o- or S9 cells) which had been pre-treated without (PBS, control) or with 5000 ng/mL rHlb (sphingomyelinase). As shown in Figure S1, sphingomyelin (SM) was present in both cell types under control conditions (PBS), however, with a higher abundance in 16HBE14o- cells compared with S9 cells. Incubation of cells with rHlb (rHlb) reduced SM abundance in S9 as well as in 16HBE14o- cells. Other lipids, like phosphatidylcholine (PC), phosphatidylserine (PS), or phosphatidylethanolamine (PEA) remained unaffected. Subsequent treatment of cells with 2000 ng/mL rHla did not generate additional effects on the SM levels (results not shown). Hence, sphingomyelinase activity of the rHlb preparation was confirmed.

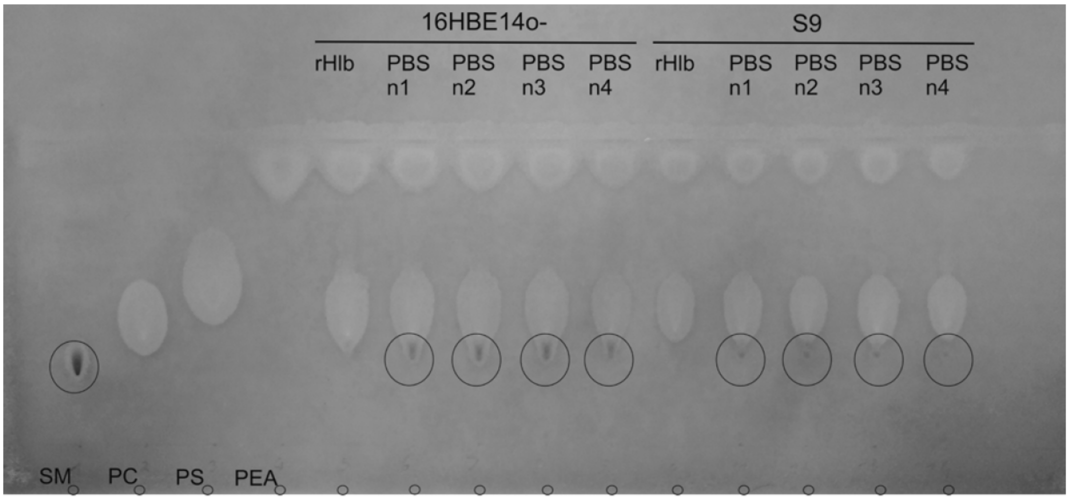

Figure S1. Incubation of cells with sphingomyelinase (rHlb) reduced sphingomyelin abundance in PMs of airway epithelial cells. Phospholipids isolated from 16HBE14o- or S9 cells were separated using high performance thin-layer chromatography. Sphingomyelin (SM), phosphatidylcholine (PC), phosphatidylserine (PS), as well as phosphatidylethanolamine (PEA) were present in the PM lipid fraction. Upon treatment of cells with recombinant *S. aureus* hemolysin B (rHlb), the abundance of SM was below the detection limit. The other lipids were not affected by such a treatment of the cells. Representative data of three independent experiments are shown in Materials and Methods.

Materials and Methods

Semi-quantitative lipid analysis by high-performance thin-layer chromatography (HPTLC). To compare SM concentrations in S9 and 16HBE14o- cells, respectively, cells were cultured on 10 cm plates to confluence, briefly washed with PBS, trypsinized, and suspended in pre-warmed (37 °C), air-equilibrated HEPES-buffered saline [1]. Cells were washed by centrifugation (2 min at 600× g) and resuspended in HEPES-buffered saline. Cell counts were determined (Cell Counter, Biozym, Oldendorf, Germany). Cells were treated with 5000 ng/mL Hlb or PBS (control) at 37 °C with gentle shaking for 2 h, respectively. Lipids

were extracted as described previously [2] using chloroform/methanol (ratio 2:1). Extracts from 6 × 10^6 cells were prepared and dissolved in 30 µL chloroform/methanol for further analysis. Phospholipids were separated by high performance thin-layer chromatography on HPTLC silica 60 gel plates (Merck, Darmstadt, Germany) using chloroform/methanol/water (65/25/4, *v*/*v*/*v*) as the mobile phase and stained using ammonium molybdate solution. Standards of sphingomyelin, phosphatidylcholine, phosphatidylserine, and phosphatidylethanolamine were run in separate lanes for identification of lipids in the samples.

References

1. Shuttleworth, T.J.; Thompson, J.L. Intracellular [Ca^{2+}] and inositol phosphates in avian nasal gland cells. *Am. J. Physiol.* **1989**, *257*, C1020–C1029.
2. Folch, J.; Lees, M.; Sloane Stanley, G.H. A simple method for the isolation and purification of total lipides from animal tissues. *J. Biol. Chem.* **1957**, *226*, 497–509.

S2A: The recombinant form of *S. aureus* Hlb does not affect [Ca^{2+}]$_i$ in airway epithelial cells per se. To test whether recombinant hemolysin B (rHlb) had a direct effect on the intracellular calcium concentration in airway epithelial cells, we used suspended and Indo-1 loaded 16HBE14o- cells and measured Ca^{2+}-dependent fluorescence before and after addition of 5000 ng/mL rHlb (rHlb), as described previously [1]. The calibration of the fluorescence signals was performed as described [2]. As shown in Figure S2A, there were no significant long term changes in [Ca^{2+}]$_i$ observed in airway epithelial cells when compared to the respective controls treated just with phosphate buffered saline (PBS).

S2B: Pre-incubation of 16HBE14o- cells with rHlb does not render the cells unable to respond to calcium influx-inducing agents other than rHla.

Treatment of sphingomyelin-containing lipid membranes with sphingomyelinase results in the generation of water-soluble phosphocholine and membrane-bound ceramides. Due to the very small headgroup of ceramides and their tendency to from regional clusters, the curvature of the membrane may change. When ceramides are selectively produced in the outer leaflet of the membrane of a liposome, this may result in local invaginations of the membrane and even fission of smaller, ceramide-enriched vesicles to the inside of the liposome [3]. It is not quite clear whether such a process may occur also in living cells when the outer lipid layer is exposed to sphingomyelinase [4]. In such a case, however, one may expect that integral plasma membrane proteins or molecules associated with the plasma membrane may be rapidly internalized by endocytosis and removed from the cell surface. In our case, such a scenario would result in a reduction in the abundance of rHla-pores in the cell membrane, which would then result in similar observations that we actually made, namely that rHla could not induce its usual cell physiological changes in the airway epithelial cells. To exclude that our results were spoiled by such a mechanism, we performed some "proof of principle" experiments, in which we pre-incubated 16HBE14o- cells with rHlb and started monitoring [Ca^{2+}]$_i$ during the next 30 min. Other than in cells that were not pre-treated with rHlb (- rHlb + rHla), the addition of rHla to rHlb-pre-treated cells (+ rHlb + rHla) did not result in any significant increases in Ca^{2+}-dependent Indo1-fluorescence (Figure S2B), as also observed in previous experiments (Figure 2). When cells were treated with calcium ionophores (ionomycin or A23187), however, there was a rapid and intense increase in Ca^{2+}-dependent Indo1-fluorescence (Figure S2B; + rHlb + Iono or + rHlb + A23, respectively). This shows that plasma membrane-associated molecules in rHlb-pre-treated 16HBE14o- cells are not internalized at a rate that renders them inactive in mediating calcium influx from the extracellular space into the cytosol. We conclude from these observations that also rHla will still be present in the plasma membrane of rHlb-pre-treated cells in sufficient amounts to potentially induce cell physiological effects. That we do not see those effects (Figures 2–4) indicates that it is indeed the assembly of functional pores that is inhibited by rHlb-mediated removal of sphingomyelin head groups in the outer membrane leaflet.

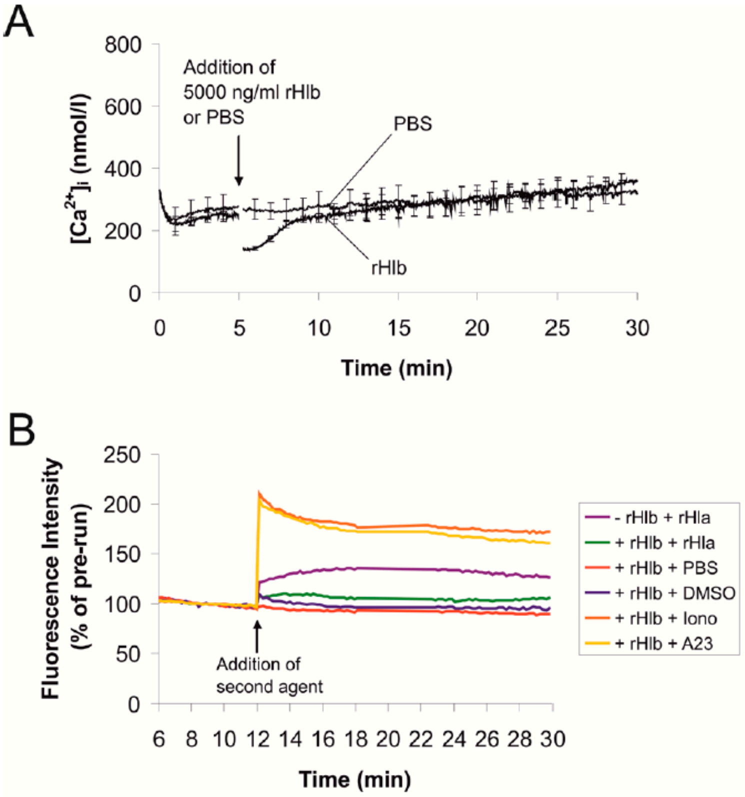

Figure S2. (**A**) Incubation of 16HBE14o- cells with sphingomyelinase (rHlb) does not readily affect $[Ca^{2+}]_i$. Means ± S.D. (at each full min) of six (PBS) or four (rHlb) experiments, respectively, are shown. (**B**) Pre-incubation of 16HBE14o- cells with rHlb does not render the cells unable to respond to calcium influx-inducing agents other than rHla. The 16HBE14o- cells were pre-incubated for 30 min with (+ rHlb) or without (-rHlb) 5,000 ng/mL rHlb during the recovery period of the Indo1-loading procedure and suspended in HEPES-buffered saline. Ca2+-dependent fluorescence of Indo-1 was recorded as described in the Materials and Methods section. One of the second agents (2000 ng/mL rHla, PBS (solvent control), DMSO (solvent control), 0.5 µmol/L ionomycin, or 5 µmol/L A23187 (both obtained from Enzo Life Sciences, Lörrach, Germany) was added as indicated. Means of two runs for each experimental design are shown.

References

1. Eichstaedt, S.; Gäbler, K.; Below, S.; Müller, C.; Hildebrandt, J.-P. Phospholipase C-activating plasma membrane receptors and calcium signaling in immortalized human airway epithelial cells. *J. Recept. Signal Transd.* **2008**, *28*, 591–612.
2. Grynkiewicz, G.; Poenie, M.; Tsien, R.Y. A new generation of Ca^{2+} indicators with greatly improved fluorescence properties. *J. Biol. Chem.* **1985**, *260*, 3440–3450.
3. Holopainen, J.M.; Angelova, M.I.; Kinnunen, P.K.J. Vectorial budding of vesicles by asymmetrical enzymatic formation of ceramide in giant liposomes. *Biophys. J.* **2000**, *78*, 830–838.
4. Chen, C.-S.; Rosenwald, A.G.; Pagano, R.E. Ceramide as a modulator of endocytosis. *J. Biol. Chem.* **1995**, *270*, 13291–13297.

S3: Sphingomyelinase (rHlb) pre-treatment prevents Hla-mediated hemolysis in sheep erythrocytes. To investigate whether the protective action of sphingomyelinase (*S. aureus* rHlb) pre-treatment of cells against deleterious actions of Hla is limited to airway epithelium or also applies to other cell systems, we conducted hemolysis assays on sheep erythrocytes in blood agar plates. Usually, a typical effect of adding rHla to the well of such an Agar plate is "complete hemolysis" of the blood cells embedded in the gel matrix around the well [1-3]. In this study, we tested the hemolytic activity of 2000 ng/mL rHla without (PBS) or with pre-incubation of sheep erythrocytes with sphingomyelinase (5000 ng/mL *S. aureus* rHlb, 1 h), respectively, on sheep blood agar plates. As shown in Figure S3, incubation of blood cells with PBS (negative control) caused no hemolysis at all (A), whereas exposure of erythrocytes to 2000 ng/mL rHla caused complete hemolysis of blood cells within the radius of 1 cm around the well (Figure S3D). Incubation of blood cells with 5000 ng/mL rHlb induced an "incomplete hemolysis" (Figure S3B). Pre-incubation of cells with 5000 ng/mL rHlb followed by treatment with 2000 ng/mL rHla caused only the "incomplete hemolysis" induced by rHlb, but no further hemolysis induced by rHla Figure S3C). Hence, pre-treatment of sheep erythrocytes with rHlb seemed to prevent rHla-mediated hemolysis.

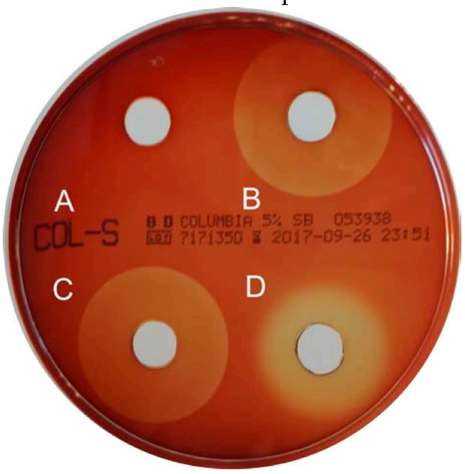

Figure S3. Pre-incubation of sheep erythrocytes with sphingomyelinase (rHlb) prevented Hla-mediated hemolysis. Hemolytic activity of rHla was observed using sheep erythrocytes in blood agar plates. PBS as a vehicle control did not cause any lytic effects (**A**), rHlb alone caused an 'incomplete hemolysis' (**B**), whereas rHla treatment resulted in a clear zone of "complete hemolysis" (**D**). Pre-incubation of erythrocytes with rHlb and subsequent incubation with rHla did not cause any additional signs of rHla-typical hemolysis (**C**). A representative example of three independent experiments is shown.

Material and Methods

Hemolysis assay. Hemolytic activity of Hla was assayed on sheep blood agar plates (Becton Dickinson, Heidelberg, Germany). Four holes (8 mm diameter) were punched into a blood agar plate. Each hole was filled with either 5000 ng/mL Hlb or PBS as negative control (150 µL, i.e., 3 × 50 µL, each). Plates were incubated at 37 °C for 1 h. Subsequently, 2000 ng/mL Hla or PBS (150 µL, i.e., 3 × 50 µL, each) as control were added and the plates were incubated over night at 37 °C. Three independent experiments with two technical replicates were carried out.

References

1. Glenny, A.T.; Stevens, M.F. *Staphylococcus* toxins and antitoxins. *J. Pathol. Bacteriol.* **1935**, *40*, 201–210.
2. Bernheimer, A.W., Schwartz, L.L. Isolation and composition of staphylococcal alpha toxin. *J. Gen. Microbiol.* **1963**, *30*, 455–468.
3 Bhakdi, S.; Muhly, M.; Füssle, R. Correlation between toxin binding and hemolytic activity in membrane damage by staphylococcal alpha-toxin. *Infect. Immun.* **1984**, *46*, 318–323.
